# Interactions between the otitis media gene, *Fbxo11*, and *p53* in the mouse embryonic lung

**DOI:** 10.1242/dmm.022426

**Published:** 2015-12-01

**Authors:** Hilda Tateossian, Susan Morse, Michelle M. Simon, Charlotte H. Dean, Steve D. M. Brown

**Affiliations:** 1 Medical Research Council, Mammalian Genetics Unit, Harwell OX11 0RD, UK; 2 Leukocyte Biology, National Heart and Lung Institute, Imperial College London, London SW7 2AZ, UK

**Keywords:** Otitis media, FBXO11, p53, TGF-β, Lung

## Abstract

Otitis media with effusion (OME) is the most common cause of hearing loss in children, and tympanostomy (ear tube insertion) to alleviate the condition remains the commonest surgical intervention in children in the developed world. Chronic and recurrent forms of otitis media (OM) are known to have a very substantial genetic component; however, until recently, little was known of the underlying genes involved. The *Jeff* mouse mutant carries a mutation in the *Fbxo11* gene, a member of the F-box family, and develops deafness due to a chronic proliferative OM. We previously reported that *Fbxo11* is involved in the regulation of transforming growth factor beta (TGF-β) signalling by regulating the levels of phospho-Smad2 in the epithelial cells of palatal shelves, eyelids and airways of the lungs. It has been proposed that FBXO11 regulates the cell's response to TGF-β through the ubiquitination of CDT2. Additional substrates for FBXO11 have been identified, including p53. Here, we have studied both the genetic and biochemical interactions between FBXO11 and p53 in order to better understand the function of FBXO11 in epithelial development and its potential role in OM. In mice, we show that *p53* (also known as *Tp53*) homozygous mutants and double heterozygous mutants (*Jf*/+ *p53*/+) exhibit similar epithelial developmental defects to *Fbxo11* homozygotes. FBXO11 and p53 interact in the embryonic lung, and mutation in *Fbxo11* prevents the interaction with p53. Both *p53* and double mutants show raised levels of pSMAD2, recapitulating that seen in *Fbxo11* homozygotes. Overall, our results support the conclusion that FBXO11 regulates the TGF-β pathway in the embryonic lung via cross-talk with p53.

## Abstract (precis)

**Summary:** Genetic interactions between *Fbxo11* and *p53* illustrate the cross-talk between the TGF-β and p53 signalling pathways in epithelial development, with implications for the underlying molecular pathology of otitis media.

## INTRODUCTION

Otitis media with effusion (OME) is the commonest cause of hearing loss in children, and has effects on language development and learning, accompanied by behavioural problems. It is the most common cause of surgery for children in the developing world, involving the insertion of tympanostomy tubes. Genetic studies in the human population demonstrate that there is a substantial genetic component to chronic OME (COME) (Casselbrant et al., 1999, 2009; Rovers et al., 2002; Daly et al., 2004; Hafren et al., 2012b), but very little is known about the underlying genes and pathways involved. However, a number of single gene mutations in mouse give rise to COME phenotypes, in some cases accompanied by a spectrum of other disorders (Rye et al., 2011a), and provide important tools for understanding the pathways and mechanisms underlying chronic middle-ear inflammatory disease.

We have studied three mouse models of COME, *Jeff* (*Jf*) (Hardisty-Hughes et al., 2006), *Junbo* (Parkinson et al., 2006) and a knockout of the *Tgif1* gene (Tateossian et al., 2013). The *Jf* and *Junbo* mutants carry mutations in the *Fbxo11* and *Evi1* genes, respectively, and all three genes  (*Fbxo11*, *Evi1* and *Tgif1*) impact upon TGF-β signalling. EVI1 is a co-repressor of SMAD3, whereas TGIF1 functions as a regulator of the TGF-β signalling pathway. The development of COME in these mutants might reflect the interplay between TGF-β and hypoxia signalling pathways (Cheeseman et al., 2011). Hypoxia is a feature of inflamed microenvironments and there is cross-talk between TGF-β and hypoxia-inducible factor-1α (HIF-1α) signalling pathways. We investigated the occurrence of hypoxia and HIF-mediated responses in *Junbo* and *Jf* mutants, and showed cellular hypoxia in the white blood cells of both the middle-ear mucosa and middle-ear lumen (Cheeseman et al., 2011).

Our previous studies of the *Jf* mutant indicate that FBXO11 is involved in controlling TGF-β signalling by regulating the levels of pSMAD2 in embryonic epithelial cells (Tateossian et al., 2009). Whereas *Jf* heterozygous mice develop deafness due to chronic proliferative otitis media (OM) (Hardisty et al., 2003; Hardisty-Hughes et al., 2006), *Jf* homozygotes display developmental epithelial abnormalities, including underdeveloped lungs (Tateossian et al., 2009)*.* The TGF-β signalling pathway is integral for normal lung development, with all three TGF-β isoforms being expressed in embryonic lung (Bartram and Speer, 2004). It has been shown that these isoforms can negatively regulate lung branching morphogenesis in early lung development (Sakurai and Nigam, 1997; Liu et al., 2000). In contrast, downregulation of TGFβRII stimulates embryonic lung branching *in vitro* (Zhao et al., 1996). SMAD2 and SMAD3 knockouts have markedly different phenotypes, with knockout of SMAD2 leading to early embryonic lethality (Weinstein et al., 1998), whereas SMAD3 knockouts survive to term but show airspace enlargement and abnormal alveolarization (Chen et al., 2005). In adult lungs, TGF-β signalling is a key driver of remodelling after injury in diseases such as asthma, idiopathic fibrosis (IPF) and chronic obstructive pulmonary disease (COPD). In addition, single nucleotide polymorphisms (SNPs) in both SMAD3 and TGFβRII have been associated with lung function changes in a recent genome-wide association study (Postma and Timens, 2006; Moffatt et al., 2010; Liao et al., 2014; Pain et al., 2014).

FBXO11 is a member of the F-box family of proteins that are the substrate-recognition components of the SCF ubiquitin-ligase complexes containing Skp1, Cullin, Rbx1/Roc1/Hrt1 RING domain protein and an F-box protein. These proteins are important regulators of many cellular processes such as DNA replication, mitosis, DNA repair, transcription, cell differentiation and cell death (Cardozo and Pagano, 2004). FBXO11 has been identified as an E3 ligase for p53, which can promote the neddylation and suppress transcriptional activity of p53 (Abida et al., 2007). FBXO11 has also been shown to target the oncoprotein BCL6 (B cell lymphoma 6) for degradation (Duan et al., 2012). The recent identification of FBXO11 as a regulator of TGF-β signalling by controlling CDT2 activity has revealed a new avenue of TGF-β regulation (Abbas et al., 2013a,b; Rossi et al., 2013). The CRL4^Cdt2^ E3 ubiquitin ligase is a known regulator of cell cycle progression and genome stability. CDT2 was found to be degraded by CUL1-FBXO11 (CRL1^FBXO11^), which results in stabilization of p21 and SET8. It has been proposed that the epithelial defects in *Jf* mice might arise from impaired SET8 levels (Abbas et al., 2013b). Because SET8 seems to promote SMAD2 de-phosphorylation, by an unknown mechanism, the high levels of pSMAD2 observed in *Jf* mutants could reflect a compromised FBXO11-CDT2-SET8 pathway (Abbas et al., 2013a).

**TRANSLATIONAL IMPACT**

**Clinical issue**

Otitis media with effusion (OME), the build-up of thick and sticky fluid in the middle ear in the absence of infection, is the most common cause of hearing loss in children. Alleviation of the condition by the insertion of a grommet – a small tube that drains fluid from the middle ear – into the tympanic membrane (eardrum) remains the commonest surgical intervention in children in the developed world. However, the mechanisms by which this procedure, known as a tympanostomy, leads to improvement of symptoms are not clear. Chronic and recurrent forms of OM are known to have a very substantial genetic component and, until recently, little was known of the underlying genes involved. The identification of mouse models of chronic OM has transformed our understanding of the genetic pathways involved and is highlighting new avenues and targets for therapeutic intervention. A number of the mouse models reported, including the mutants *Jeff*, *Junbo* and *Tgif1*, implicate the role of the transforming growth factor-beta (TGF-β) signalling pathway in predisposition to chronic otitis media, and highlight the role of hypoxia, a feature of an inflammatory microenvironment. The interplay between the TGF-β signalling pathway and other signalling pathways in OME requires further investigation.

**Results**

In this study, the authors examined the interaction between FBXO11, a member of the F-box family of proteins that is mutated in the *Jeff* mouse model, and p53, a putative substrate of FBX011. They previously showed that FBXO11 controls levels of phosphorylated SMAD2 to regulate TGF-β signalling during epithelial development. Here, they demonstrate that, like *Fbxo11* (*Jeff*) homozygotes, *p53* homozygote mutants develop epithelial developmental abnormalities. They also investigated the genetic and biochemical interaction of *Fbxo11* with *p53*. Mice heterozygous for both genes, *Jf/+ p53/+*, exhibit similar epithelial developmental defects to *Jeff* and *p53* homozygotes. In mouse embryonic lungs, FBXO11 co-immunoprecipitates with p53, and p53 is neddylated (conjugation of the ubiquitin-like protein NEDD8) by FBXO11. Moreover, raised levels of phosphorylated SMAD2 are detected in *p53* homozygote and double heterozygote (*Jf/+ p53/+*) mutant embryonic lungs. Taken together, these results support the hypothesis that FBXO11 regulates the TGF-β pathway via cross-talk with p53.

**Implications and future directions**

This study highlights the role of FBXO11 and its interacting partner, p53, in epithelial development. The interactions between TGF-β and p53 signalling might be mimicked in OME, potentially providing new insight into the underlying molecular mechanisms of hearing loss. Thus, it will be important to translate our findings of the interaction of FBXO11 and p53 signalling in lung development to the epithelial cells of the middle ear. This will further elaborate the pathways and mechanisms underlying chronic middle-ear inflammatory disease and pave the way for new treatments to circumvent the need for surgical intervention.

In parallel, it is also known that the TGF-β and p53 pathways cooperate and a number of genes are under the joint control of p53 and SMADs (Atfi and Baron, 2008). For example, p53 physically interacts with SMAD2 *in vivo* and both are recruited at distinct cis-regulatory elements on a common target promoter, leading to synergistic activation of transcription (Cordenonsi et al., 2003). Mutant p53 can attenuate the ability of TGF-β1 to induce the expression of genes encoding p21, PAI-1 (plasminogen activator inhibitor), MMP2 (matrix metalloproteinase) and SM22 (smooth muscle specific 22 kDa protein) (Kalo et al., 2007). It has also been found that Ras signalling induces p53 N-terminal phosphorylation, enabling the interaction of p53 with activated SMADs and subsequent recruitment of the complex to specific TGF-β responsive target promoters (Cordenonsi et al., 2007).

This extensive cross-talk between TGF-β and p53 signalling pathways suggested a potential role for p53 signalling in epithelial development. Examining the interactions between TGF-β and p53 signalling pathways in epithelial development will help inform us about the molecular mechanisms impacting on epithelial function in the middle ear. We hypothesised that embryonic epithelial development might be controlled by FBXO11, through its regulation of the TGF-β pathway, in cooperation with p53. We therefore studied in detail the genetic and biochemical interactions of *Fbxo11* and *p53* (also known as *Tp53*) in developing mouse lungs, an organ that has similar properties to the middle ear (Takahashi, 2001) and for which we have previously demonstrated that FBXO11 has an important role in modulating TGF-β signalling. Our results support the conclusion that FBXO11 regulates TGF-β signalling in murine embryonic lung through interactions with the p53 pathway.

## RESULTS

### Developmental abnormalities in *p53*-deficient mice

It has been reported that a mutation in the *p73* gene, a close relative of *p53*, results in the development of OM (Yang et al., 2000). We have tested *p53*-knockout mice for hearing loss by clickbox and, in addition, we have analysed histological sections of the bullae of the mutant mice. We did not detect any difference in the hearing ability between *p53* mutant mice and wild types at the age of 2 months. Assessing the bulla sections of p53 mutant heterozygote and homozygote mice revealed no histological changes in the middle ear of either genotype.

**Fig. 1.**
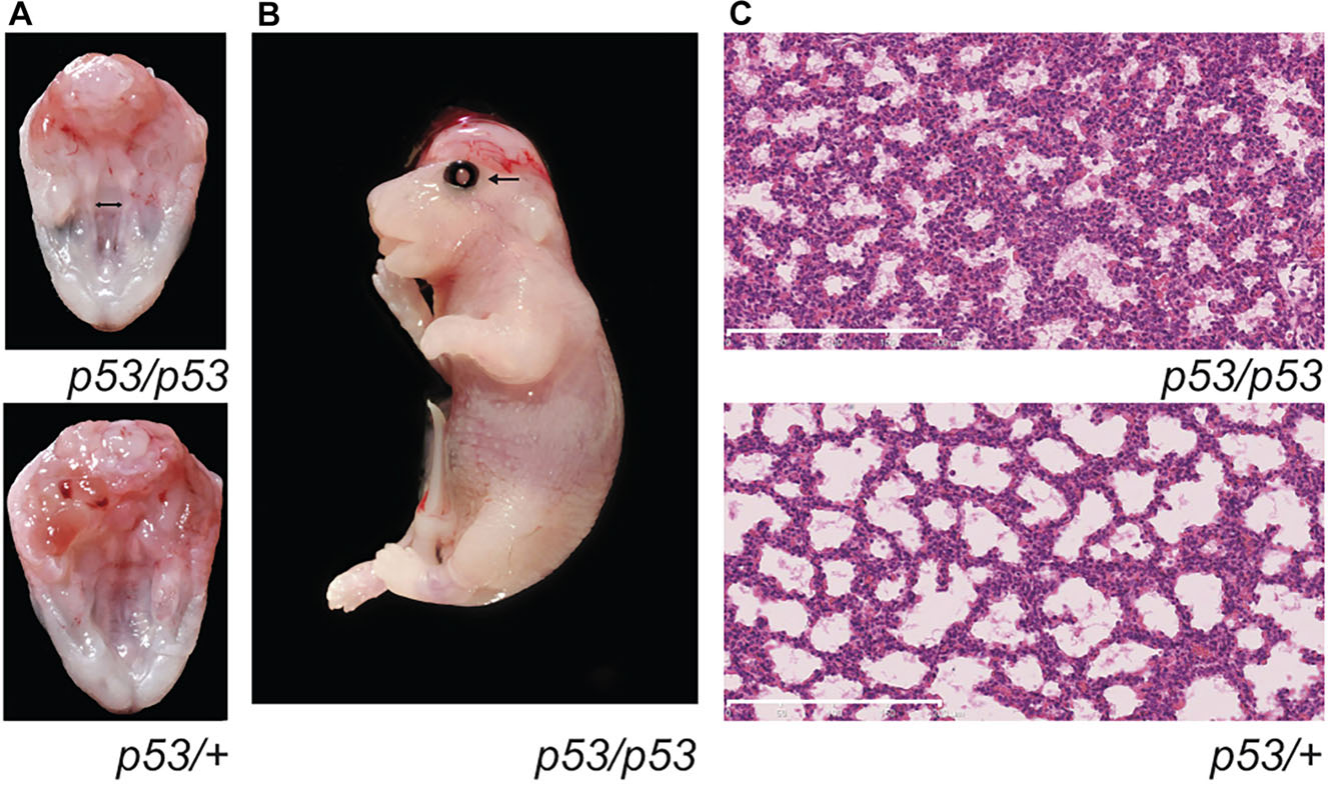
**Phenotype of *p53*-knockout mice.** (A) View of the palate of an E18.5 *p53* homozygote embryo (*p53/p53*) and a control sibling (*p53/+*) embryo. The double-headed arrow indicates the cleft palate. (B) Photograph of a *p53/p53* E18.5 embryo with the eyelids open (arrow) phenotype. (C) Sections through the lungs of an E18.5 *p53* homozygote embryo (*p53/p53*) and a control sibling (*p53/+*) embryo, showing the homozygous lung phenotype. Note the reduced number and width of airspaces in the affected *p53/p53* homozygote (see also Fig. 2). Scale bars: 200 μm.

Mice deficient for p53 on a mixed genetic background have been reported as developmentally normal (Donehower et al., 1992). However, outcrossing the mice on a 129/sv background results in embryonic lethality due to defects in neural tube closure (Sah et al., 1995), and additional developmental abnormalities are reported on a 129/Ola background (Armstrong et al., 1995). Owing to the variable phenotypes observed on different backgrounds, we first undertook a detailed investigation of embryogenesis in our p53 mutant colony. We noted that, in p53 mice maintained on a C57BL/6J background, a significant proportion of homozygotes died before weaning. The surviving homozygotes composed 14.3% (25/175) of the mice from heterozygote intercrosses, less than the expected 25% [χ^2^=10.71429 *P*=0.0011, degrees of freedom (d.f.)=1]. Only three out of the 25 adult homozygote mice were female. At embryonic day 18.5 (E18.5), four out of ten homozygote embryos had developmental abnormalities. All four affected embryos had severely abnormal lung architecture; in addition, one had eyelids open and exencephaly and two had cleft palate (Fig. 1) – phenotypes that hitherto have not been reported in *p53*-deficient mice. Of these four embryos, three were female and one male. This is consistent with previous reports that some developmental defects in *p53*-deficient mice, such as exencephaly, are known to be female-specific (Sah et al., 1995).

**Fig. 2.**
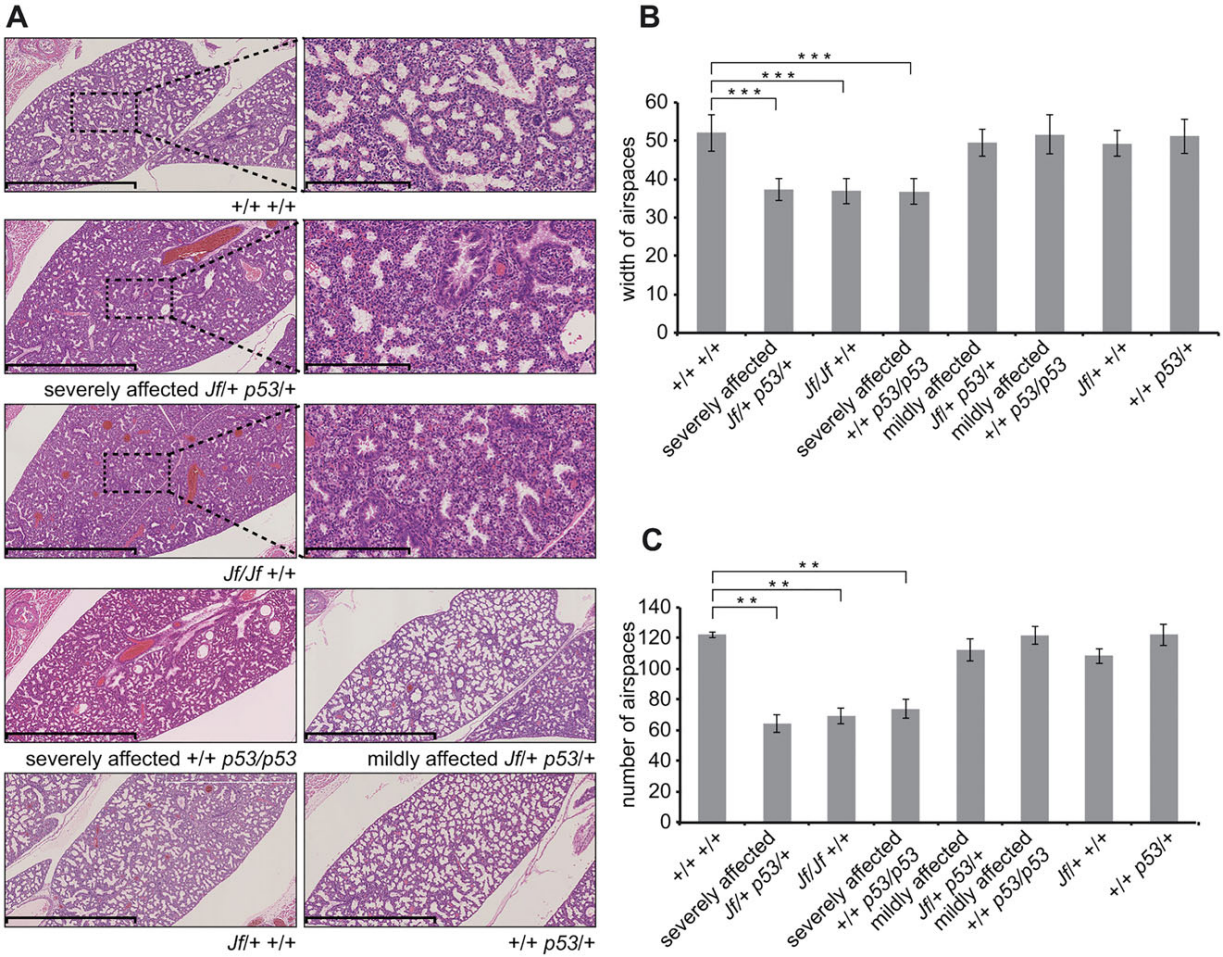
**Characterization of lung phenotypes in *p53/p53* homozygotes and *Jf/+ p53/+* double heterozygote mice.** (A) Haematoxylin- and eosin-stained sections through E18.5 embryo lungs showing lung phenotypes from a variety of genotypes. Scale bars: 1 mm and 200 μm. (B) Comparisons of the width of 20 airspaces from three different regions for three embryos of each genotype. (C) Comparisons of the number of airspaces for three 4×10^5^-μm^2^ regions for three embryos of each genotype. The sections were taken at random for the three embryos from each genotype. Bars: standard error of mean (s.e.m.). **P<0.01 and ***P<0.001.

To analyze the lung phenotype of the embryos, we measured the width and counted the number of the airways as described previously (Yates et al., 2013). The E18.5 p53 homozygote lungs showed significantly reduced airspace width (*P*=4.33E-06; Fig. 2A,B) and a significantly reduced number of airspaces compared to wild-type littermates (*P*=0.0024; Fig. 2A,C). The remaining six embryos had less disrupted lung histology and a milder lung phenotype. These phenotypic features closely resemble those observed in *Jf* homozygotes (Hardisty-Hughes et al., 2006; Tateossian et al., 2009) and likely account for the perinatal mortality of *p53*-deficient mice.

### *Fbxo11* and *p53* interact genetically

Given the similarities between *Fbxo11* and *p53* phenotypes, and our hypothesis regarding potential interactions between FBXO11 and p53, we tested for genetic interactions between these two loci. In our previous study, intercrosses of *Jf* heterozygotes and *p53* homozygotes on a C57BL/6J background produced compound heterozygotes, most of which survived (Tateossian et al., 2009). However, in this study, using *Jf* mice on a mixed C3H/HeH-C57BL/6J background lead to reduced survival of compound heterozygotes (19/146, χ^2^=11.1872 *P*=0.0008, d.f.=1). The average weight of the compound mutants was the same as the average weight of *Jf* heterozygotes (Fig. S1).

Given that our previous work examining interactions between FBXO11 and TGF-β signalling had focused on the lungs as one of the most severely affected organs (Tateossian et al., 2009), we chose to investigate interactions between FBXO11 and p53 in the same organ. At E18.5, half of the double heterozygotes (*Jf*/+ *p53*/+) examined (5/10) had severely affected lungs and cleft palate, a phenotype similar to that of *Jf* homozygote mice (Tateossian et al., 2009), mice heterozygous for both *Jf* and *Smad2* (Tateossian et al., 2009), and some *p53* homozygote mice. The average width (*P*=0.00001; Fig. 2A,B) and number (*P*=0.0019; Fig. 2A,C) of airspaces in severely affected double heterozygotes was significantly lower than in wild types, recapitulating the lung phenotype of the *Jf/Jf* embryos and of the affected *p53/p53* embryos, indicating a genetic interaction between *Fbxo11* and *p53*.

Surviving double heterozygotes looked phenotypically normal and we therefore intercrossed them. *Jf/Jf p53/p53* and *Jf/Jf* *p53/+* embryos were examined at E18.5, E17.5 and E16.5. At E16.5, we did not find any *Jf/Jf* *p53/p53* homozygotes, indicating that they die before E16.5, and we found one *Jf/Jf* *p53/+* embryo of normal size (χ^2^=2.172414 *P*=0.140506, d.f.=1), suggesting that *Jf/Jf p53/+* mutants die after E17.5 (Table S1).

### FBXO11 and p53 are in a complex with CUL1 in the embryonic mouse lung

In order to assess the biochemical interactions between FBXO11 and p53 in developing epithelia, we focused our analysis on the lung. Because of to its small size, protein analysis, such as immunoprecipitation, of middle-ear epithelia is difficult. However, both the middle ear and the lungs have substantial similarities in structure and function (Takahashi, 2001).

**Fig. 3.**
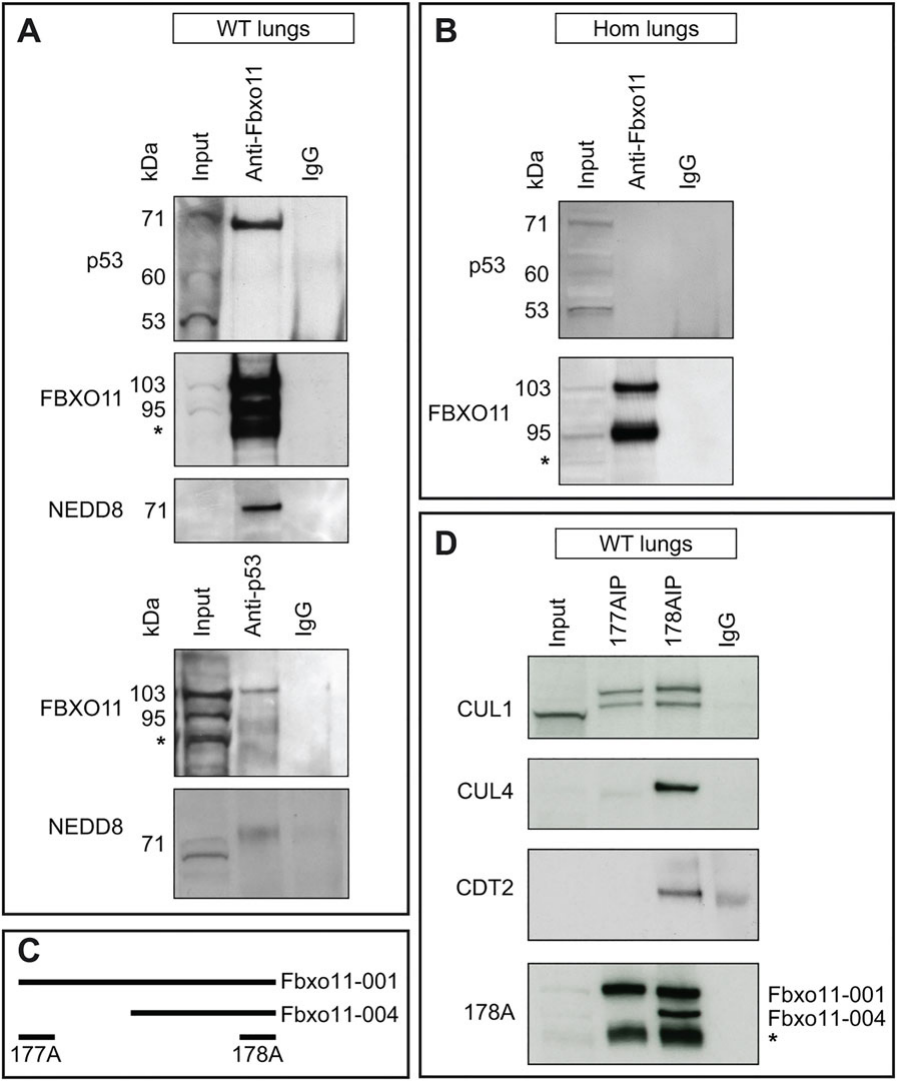
**Interaction of FBXO11 with p53.** (A) Protein lysate from E15.5 wild-type embryonic lungs was used for immunoprecipitation using an anti-FBXO11 (top, second and third panel) or an anti-p53 (fourth and fifth panel) antibody. Normal rabbit IgG was used as a control (IgG). Western blots were probed with antibodies as indicated: p53 (panel 1); FBXO11 (178A which detects both large and small isoforms of FBXO11, 103 and 95 kDa; panel 2 and 4); NEDD8 (panel 3 and 5). A degradation band observed with FBXO11 is indicated by an asterisk. (B) Protein lysate from E15.5 *Jeff* homozygote (Hom) lungs was used for immunoprecipitation using an anti-FBXO11 antibody (178A). Normal rabbit IgG was used as a control (IgG). The top panel was probed with an anti-p53 antibody, the bottom with an anti-FBXO11 antibody (178A). (C) Alignment of the fragments of FBXO11 protein used to produce the 177A and 178A antibodies for FBXO11. The epitope recognized by 177A maps to a region between residue 1 and 50 of human FBXO11, which is exclusive to the large isoform of mouse FBXO11 (Fbxo11-001). The epitope recognized by 178A maps to a region between residue 877 and 927 of human FBXO11 and is found in both mouse isoforms (Fbxo11-001 and 004). (D) Protein lysate from E15.5 wild-type (WT) embryonic lungs was used for immunoprecipitation using two anti-FBXO11 antibodies: 177A, which detects the large isoform of mouse FBXO11 (Fbxo11-001, 930 aa, 103 kDa), and 178A, which detects both the large and small isoforms (Fbxo11-001, 930 aa, 103 kDa and Fbxo11-004, 855 aa, 95 kDa). The blots were probed with anti-CUL1, -CUL4, -CDT2 and 178A antibodies. A degradation band of FBXO11 is indicated by an asterisk.

In our previous study, we were not able to demonstrate a biochemical interaction between *p53* and *Fbxo11* in lung (Tateossian et al., 2009). Given the genetic interaction reported here, we substantially modified our approach to again look for a biochemical interaction, including the use of alternative antibodies. Total protein from E15.5 wild-type lungs was immunoprecipitated with an antibody that detects both the short and long forms of FBXO11, and we found that p53 does coimmunoprecipitate with FBXO11 (Fig. 3A, top and second panel). The size of the p53 band (71 kDa) suggested that the protein is modified. It has been shown that FBXO11 promotes the neddylation of p53 at Lys320 and Lys321 (Abida et al., 2007). NEDD8 is 9 kDa and therefore the expected size of FBXO11-neddylated p53 is 71 kDa. By employing an anti-NEDD8 antibody, we demonstrated that p53 is neddylated when complexed with FBXO11 in the E15.5 mouse lung (Fig. 3A, third panel) and, in addition, the anti-NEDD8 antibody detects a band with the size of about 71 kDa in the p53 immunoprecipitate (Fig. 3A, bottom panel). We also undertook reverse immunoprecipitation using a rabbit anti-p53 antibody. We found that only the larger isoform of FBXO11 (103 kDa) coimmunoprecipitates with p53 in the mouse lung (Fig. 3A, fourth panel). To test whether the *Jf* mutation prevents the association of FBXO11 with p53, we conducted an immunoprecipitation with total protein isolated from E15.5 *Jf* homozygote lungs. The anti-FBXO11 antibody precipitated FBXO11 from the mutant embryonic lungs (Fig. 3B, second panel) but not p53 (Fig. 3B, first panel).

F-box proteins are one of the core subunits of the SCF complex. Cullins are the other important unit providing a scaffold for the complex. We sought to determine the interaction of FBXO11 with CUL1 and CUL4 in E15.5 lungs. We used two anti-FBXO11 antibodies: antibody 177A, which recognises only the larger FBXO11 isoform, and 178A, which detects both isoforms (Fig. 3C). As expected, the 178A antibody recognized a single 103 kDa band in the 177A immunoprecipitation corresponding to the large FBXO11 isoform (ENSMUSP00000005504), whereas, in the 178A immunoprecipitation, it recognized two bands, the large 103 kDa isoform but also the small 95 kDa isoform (ENSMUSP00000130379) (Fig. 3D, bottom panel). Surprisingly, we found that CUL4 was coimmunoprecipitated with the 178A anti-FBXO11 antibody but not 177A, suggesting that the small isoform of FBXO11 is in a complex with CUL4 (Fig. 3D, second panel). It has been reported that the short isoform of FBXO11 binds CDT2 and plays the key role in CDT2 degradation in the FBXO11-CDT2-SET8 pathway (Abbas et al., 2013b). CDT2 also binds to CUL4 through the cullin 4-RING ubiquitin ligase. We found that CDT2 was also coimmunoprecipitated with the 178A anti-FBXO11 antibody (Fig. 3D, third panel). It is possible that the short isoform of FBXO11 is binding to CDT2 in mouse lung tissue as part of this complex and, as a consequence, CUL4 is coimmunoprecipitated. An anti-CUL1 antibody detected two bands in both immunoprecipitations, using 177A and 178A, indicating that the larger isoform, which interacts with p53, is in an SCF complex with CUL1 (Fig. 3D, top panel).

### Structural analysis demonstrates possible interactions between FBXO11 and p53

**Fig. 4.**
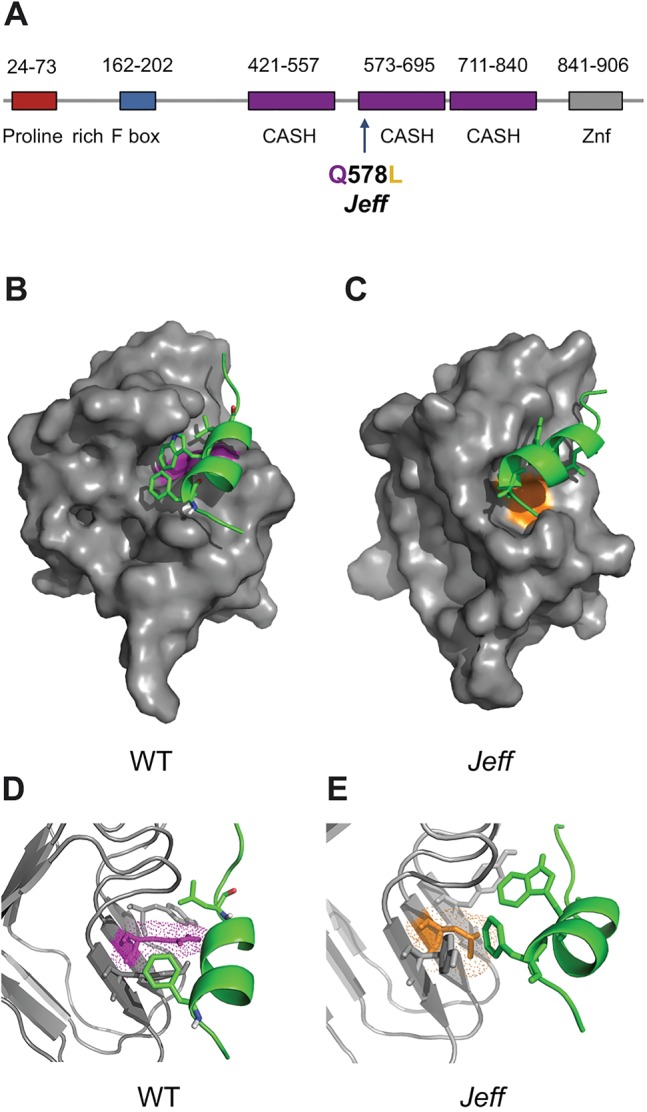
**Predicted structure** **of the FBXO11-p53 complex.** (A) Localization of the *Jeff* mutation (Q578L) in the predicted protein structure of FBXO11 on Ensembl. CASH, carbohydrate-binding proteins and sugar hydrolysis; Znf, zinc finger domain. (B-E) Cartoon representation of p53 (green) docked in the FBXO11 (grey) hydrophobic cleft to form the FBXO11-p53 complex. The wild-type (WT) complex is on the left with the wild-type residue (Q) coloured purple (B,D). The putative mutated complex found in the *Jeff* mutation is indicated on the right with the mutated amino acid (L) coloured orange (C,E). The mutated complex has an extended and distorted binding site, possibly contributing to the loss of binding to p53. Figure produced using The PyMOL Molecular Graphics System, Version 1.5.0.4 Schrödinger, LLC.

Following the demonstration that the *Jf* mutation interferes with the interaction between FBXO11 and p53, we sought to better understand the structural relationship between the FBXO11-p53 protein-protein interaction. FBXO11 is a 930-amino-acid F-box protein that contains a proline-rich domain and the F-box domain at the N-terminus, three central CASH (carbohydrate-binding proteins and sugar hydrolysis) domains and a zinc-finger domain at the C-terminus. The *Jf* mutation, a non-conservative glutamine-to-leucine change (Hardisty-Hughes et al., 2006), is located at the beginning of the second CASH domain (Fig. 4A). CASH domains are found in over 1000 proteins and consist of repeats of approximately 7 to 11 right-handed β-helixes (Ciccarelli et al., 2002). Structural analysis demonstrates the possible biochemical basis for FBXO11 interaction with the transactivation domain of p53. Utilising protein docking predictions, we found that the second CASH domain forms a deep hydrophobic cleft into which the α-helix that is present in the N-terminus transactivation domain of p53 can bind (Fig. 4B,D). A similar lock-and-key mechanism is seen with the MDM2 (murine double minute 2)-p53 interaction (Kussie et al., 1996; Moll and Petrenko, 2003). MDM2 is one of the regulators of p53 and acts as an E3 ligase to promote p53 ubiquitination and neddylation. MDM2 interacts with the transactivation domain of p53 (Kussie et al., 1996) and ubiquitinates and neddylates amino acids at the C-terminus of p53. FBXO11 is also known to promote the neddylation of amino acids localized in the C-terminus of p53 (Abida et al., 2007). Our structural analysis predicted a possible interaction of FBXO11 with the transactivation domain of p53, similar to the one seen with MDM2. The *Jf* mutation is centrally located within the hydrophobic cleft. We therefore hypothesise that the non-polar leucine might disrupt hydrogen bonds within the binding site (Fig. 4C,E), weakening FBXO11's affinity for p53.

### FBXO11 neddylates p53 in the developing lung

**Fig. 5.**
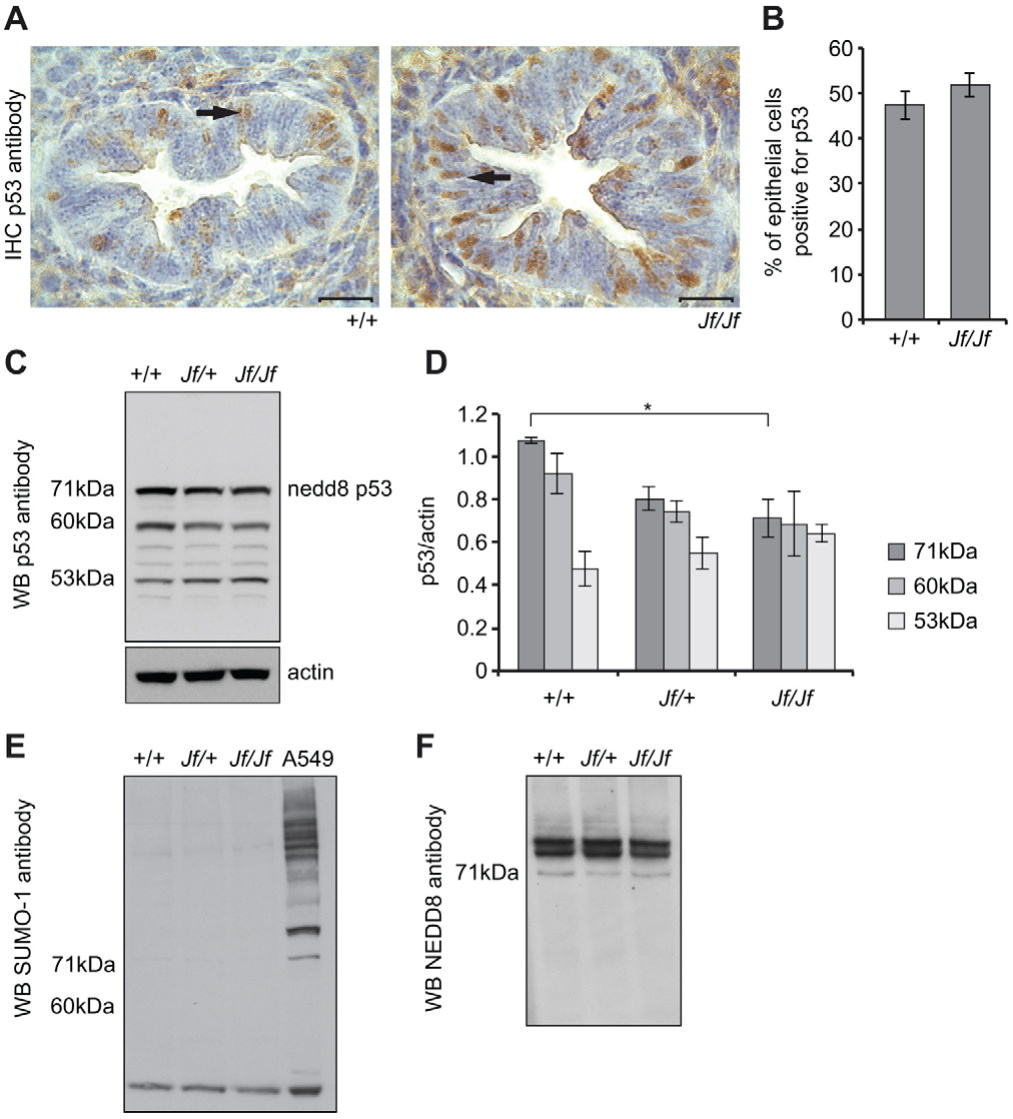
**Expression of p53 in embryonic lungs.** (A) Sections through E15.5 wild-type (+/+) and *Jeff* homozygote *(Jf/Jf*) embryonic lungs stained with an anti-p53 antibody (brown stain). The nuclei were counterstained with haematoxylin. In both the wild-type and mutant epithelial cells of the airways, p53 is predominantly nuclear (arrow). Scale bars: 20 μm. (B) Comparisons of the percentage of cells positive for p53 in wild-type and homozygote airways. In total, 238 (+/+) and 211 *(Jf/Jf*) epithelial cells were counted from the airways of different regions of three embryos for each genotype. Bars: standard error of mean (s.e.m.). No significant difference was found. (C) Equal amounts of protein lysates from E15.5 wild-type (+/+), heterozygous (*Jf/*+) and homozygous (*Jf/Jf*) lungs were subjected to PAGE, transferred and probed with an anti-p53 antibody. The antibody detected three main bands (see Results): the 53 kDa band represents unmodified or acetylated/phosphorylated p53; the 60 kDa band likely represents monoubiquitinated p53; and the 71 kDa band corresponds to neddylated p53. (D) Comparisons of p53 levels across genotypes after normalizing with an anti-actin antibody. The results presented in the graph are from three independent experiments. Bars: s.e.m. *P<0.05. (E) Equal amounts of protein lysates from E15.5 wild-type (+/+), heterozygous (*Jf/+*) and homozygous (*Jf/Jf*) lungs were subjected to PAGE, transferred and probed with an anti-SUMO-1 antibody. The antibody did not detect bands with the size of 60 kDa. A549 cell lysate was used as a positive control for the anti-SUMO-1 antibody. (F) Equal amounts of protein lysates from E15.5 wild-type (+/+), heterozygous (*Jf/+*) and homozygous (*Jf/Jf*) lungs were subjected to PAGE, transferred and probed with an anti-NEDD8 antibody. The antibody detected at least three bands, one of which was 71 kDa. WB, western blot.

It has previously been shown that NEDD8 conjugation to the C-terminus of p53 does not change the subcellular localization of p53 in H1299 cells (Abida et al., 2007). Immunohistochemical analysis of p53 in wild-type and *Jf/Jf* embryonic lungs showed that p53 is predominantly localised in the nucleus of the epithelial cells of the airways, suggesting that the mutation in FBXO11 does not alter localization of p53 (Fig. 5A,B). Western blot analysis with anti-p53 antibody on wild-type and mutant lung tissue detected three main bands at approximately 53, 60 and 71 kDa (Fig. 5C). It is well known that the p53 protein is post-translationally modified in several ways. The 53 kDa band probably corresponds to unmodified p53 or phosphorylated/acetylated p53. The 60 kDa band could be either monoubiquitinated (ubiquitin is 8.5 kDa) or monosumoylated (SUMO proteins are 12 kDa) p53. However, an anti-SUMO-1 antibody recognized some sumoylated proteins but not of the same size (60 kDa) in embryonic lung lysates (Fig. 5E). This result suggests that the 60 kDa protein could correspond to the monoubiquitinated p53. Probing the western blot with an anti-NEDD8 antibody identified at least three proteins sized between 71 and 90 kDa that are neddylated in lung lysates, including a 71 kDa band (Fig. 5F). Two proteins are known to promote p53 neddylation, MDM2 and FBXO11. Three lysine residues in the C-terminus of p53 are required for neddylation of p53 by MDM2 (Xirodimas et al., 2004; Xirodimas, 2008) and two for neddylation by FBXO11 (Abida et al., 2007). Given the size of NEDD8 (9 kDa), the FBXO11 interaction with a 71 kDa modified form of p53 detected by us and that the anti-NEDD8 antibody recognized a 71 kDa band in the p53 pulldown (see above), we conclude that the 71 kDa band corresponds to neddylated p53. We observed decreased levels of neddylated p53 in *Jf/Jf* lungs compared to wild types (*P*=0.016) (Fig. 5C,D), supporting the conclusion that FBXO11 neddylates p53 in wild-type developing lungs. Although there was a significant reduction in neddylated p53 in *Jf/Jf* homozygotes, there were no significant changes in other forms of p53 in the homozygous mutant. Neddylated p53 protein levels were also significantly reduced in E15.5 *Jf/+ p53/+* embryonic lungs compared to wild type. Interestingly this reduction was only observed in the severely affected double heterozygotes (where cleft palate was also present) (Fig. S2). Our data strongly suggests that FBXO11 regulates the activity of p53 in the embryonic lung.

### Levels of pSMAD2 in *p53/p53* mutant and *Jf/+ p53/+* double-mutant embryonic lungs

**Fig. 6.**
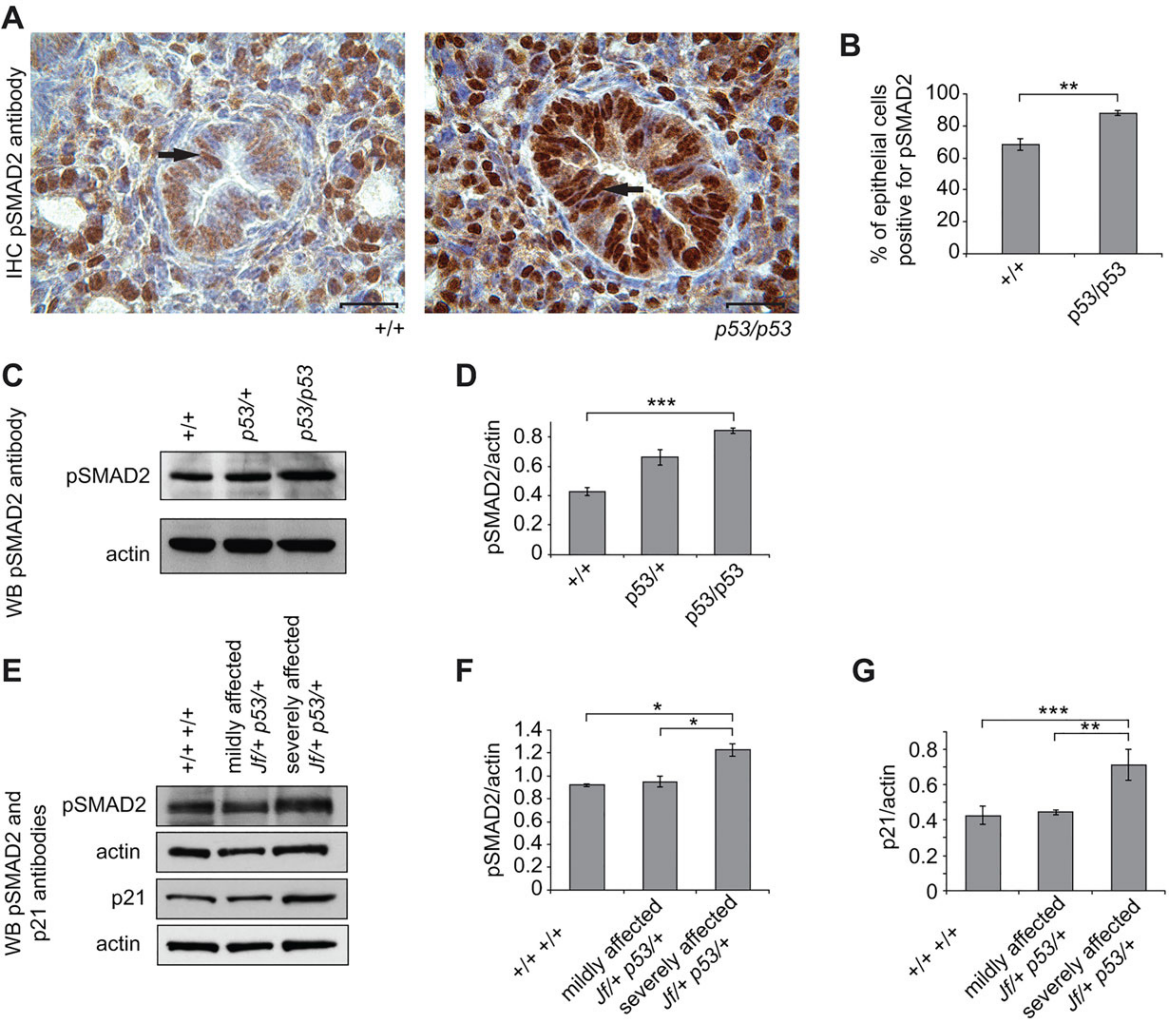
**Expression of pSMAD2 and p21 in embryonic lungs.** (A) Sections through E15.5 wild-type (+/+) and homozygous *p53* (*p53/p53*) embryonic lungs stained with an anti-pSMAD2 antibody (brown stain). The nuclei were counterstained with haematoxylin. In both wild-type and mutant epithelial cells of the airways, pSMAD2 is predominantly nuclear (arrow). Scale bars: 20 μm. (B) Comparisons of the percentage of cells positive for pSMAD2 in wild-type (+/+) and *p53* homozygote (*p53/p53*) airways. In total, 260 epithelial cells in wild types and 256 epithelial cells in *p53* homozygotes were counted from airways from different regions for three embryos from each genotype. (C) Equal amounts of protein lysates from E15.5 wild-type (+/+), heterozygous (*p53/*+) and homozygous (*p53/p53*) lungs were subjected to PAGE, transferred and probed with an anti-pSMAD2 antibody. The antibody detected one main band at about 55 kDa. (D) Comparisons of pSMAD2 levels across genotypes after normalizing with an anti-actin antibody. The results presented in the graph are from three independent experiments. (E) Equal amounts of protein lysates from E15.5 wild-type (+/+ +/+) and mildly and severely affected double-mutant (*Jf/+ p53/+*) lungs were subjected to PAGE, transferred and probed with anti-pSMAD2 and -p21 antibodies. (F,G) Comparisons of pSMAD2 (F) and p21 (G) levels across genotypes. Data represents the analysis of four individual embryos from wild-type (+/+ +/+), and mildly and severely affected double heterozygote (*Jf/+ p53/+*) lungs (based on the histological observation, without and with cleft palate, respectively) after normalizing with an anti-actin antibody. Bars: standard error of mean (s.e.m.). *P<0.05, **P<0.01 and ***P<0.001. WB, western blot.

We reported that FBXO11 modulates the TGF-β signalling pathway in the developing mouse by regulating SMAD2 activity (Tateossian et al., 2009). To determine whether pSMAD2 is also upregulated in embryonic p53 homozygotes, we performed immunohistochemistry. Because the intensity was different between the wild-type and the mutant tissues, we quantified the staining by counting the epithelial cells with nuclear stain in the airways. The percentage of pSMAD2-positive epithelial cells was significantly higher in *p53/p53* lungs, 88% (226/256), compared to wild types, 68% (181/260) (*P*=0.0012) (Fig. 6A,B). In addition, this increase was quantified by western blotting (*P*=0.0002) (Fig. 6C,D).

We also examined the levels of pSMAD2 in *Jf/+ p53/+* double-mutant E15.5 embryonic lungs. We analyzed embryonic lung lysates from mildly and severely affected double mutants. We detected a significant increase in the levels of pSMAD2 in severely affected double mutants compared to wild types (*P*=0.0291) and compared to mildly affected *Jf*/+ *p53*/+ embryos (*P*=0.0223) (Fig. 6E,F).

### Differences in the levels of p21 and PAI-1 in wild-type and *Jf-*mutant tissues

**Fig. 7.**
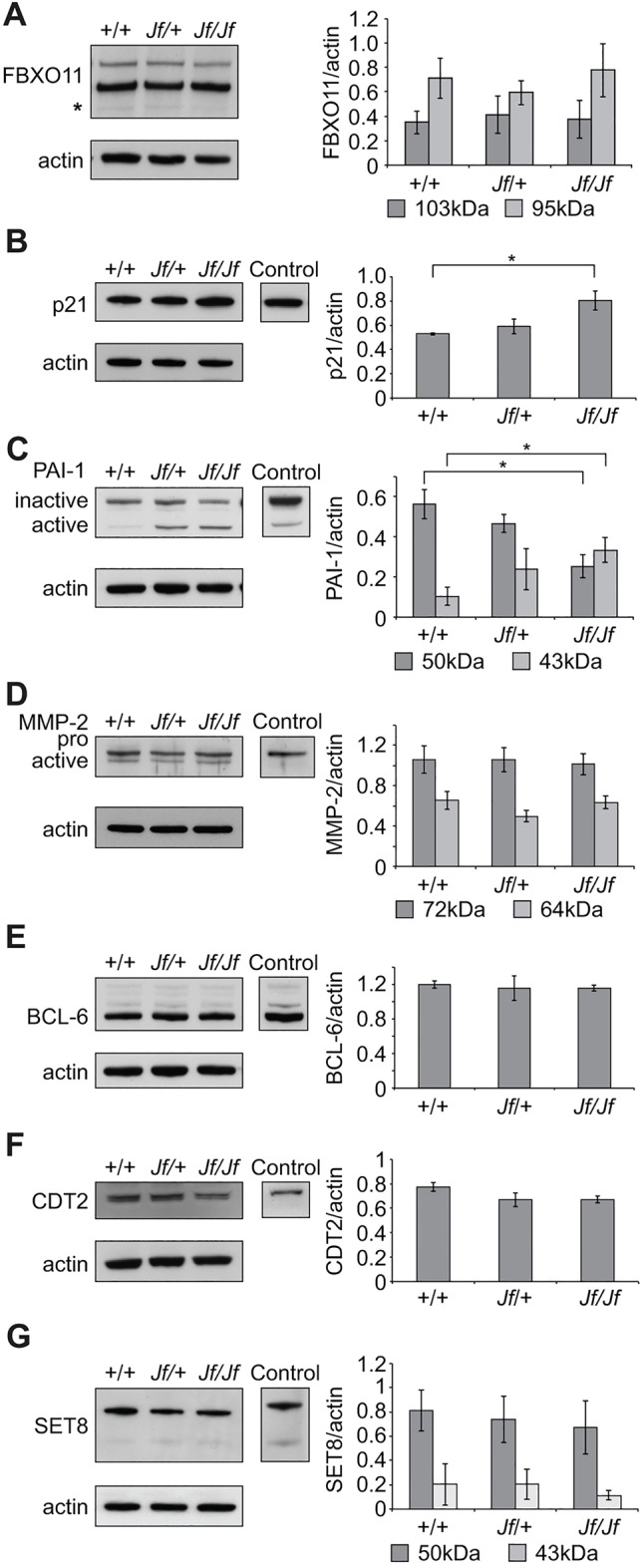
**Protein expression analysis of E15.5 wild-type** **(+/+), heterozygote (*Jf/+*) and homozygote (*Jf/Jf*) lungs.** Equal amounts of protein lysates were subjected to PAGE, transferred and probed with antibodies against FBXO11 (A), p21 (B), PAI-1 (C), MMP-2 (D), BCL-6 (E), CDT2 (F) and SET8 (G). RAW 264.7 whole-cell lysate was used as a positive control for all of the antibodies except for MMP2, where middle ear effusion lysate was used, and for CDT2, where adult mouse spleen lysate was used. Graphs show comparisons of the western blots for each antibody after normalizing with an anti-actin antibody. The results presented in the graphs are from three independent experiments for all the antibodies except for p21, where four independent experiments were used to present the data. Bars: standard error of mean (s.e.m.). *P<0.05.

To investigate further the relationship between FBXO11 and p53 in the developing mouse, we wished to assess whether FBXO11 might inhibit the activity of p53 in the developing lung. First, we examined FBXO11 levels in both *Jf* and *p53* wild-type and mutant tissues. No significant difference was seen between the wild-type and *Jf/Jf* lungs for either of the two FBXO11 isoforms (Fig. 7A). We also did not detect significant differences in the levels of either FBXO11 isoform between wild-type and mutant *p53* lungs (Fig. S3A).

Next, we investigated levels of a number of proteins that are regulated by both pSMAD2 and p53. These included p21, PAI-1 and MMP2. We detected significantly elevated levels of p21 in *Jf* homozygote lungs compared to wild-type tissue (*P*=0.0262) (Fig. 7B). We observed a significant reduction in the protein levels of the inactive form of PAI-1 (50 kDa) (*P*=0.0251) and a significant increase in the level of active PAI-1 (43 kDa) (*P*=0.0486) in *Jf/Jf* lung lysate compared to wild type (Fig. 7C). We did not detect a difference in the protein levels of either the pro-form (72 kDa) or the active form (64 kDa) of MMP2 between wild-type and mutant tissues on western blots (Fig. 7D). In contrast, protein levels of p21, PAI-1 and MMP-2 were unchanged in *p53/p53* embryonic lungs compared to littermate lungs (Fig. S3B-D). However, we observed a significant increase in levels of p21 protein in severely affected *Jf/+ p53/+* double mutants compared to wild type (*P*=0.0004) and to mildly affected embryos (*P*=0.0093) (Fig. 6E,G).

### No difference in protein levels of BCL6, CDT2 and SET8 in wild-type and mutant *Jf* and *p53* tissues

An additional avenue that we wished to investigate was whether FBXO11 might regulate the ubiquitin-mediated degradation of BCL6. FBXO11 has been found to be mutated in multiple diffuse large B-cell lymphoma cell lines and this inactivation correlates with increased levels and stability of BCL6 (Duan et al., 2012). However, we did not detect any significant difference in the protein levels of BCL6 in wild-type or *Jf-*mutant lysates (Fig. 7E). It seems that, in the mouse embryonic lung, FBXO11 does not control the degradation of BCL6. Similarly, there was no difference in the levels of BCL6 between wild-type and *p53*-mutant embryonic lungs (Fig. S3E). Finally, given the known role of FBXO11 in regulating TGF-β signalling through the degradation of CDT2 and the consequent stabilization of the substrates SET8 and p21 (Abbas et al., 2013b; Rossi et al., 2013), we examined protein levels of CDT2 and SET8 in *Jf* and *p53* mutants. We found no significant difference in the steady-state levels of either CDT2 or SET8 between wild-type and *Jf-* or *p53*-mutant tissues (Fig. 7F,G and Fig. S3F,G).

## DISCUSSION

In order to better understand the pathobiology of OM, a variety of genetic studies have been undertaken to identify loci that contribute to susceptibility to COME. A number of candidate-gene association studies have been reported (Hafren et al., 2012a). One such study demonstrated significant association with FBXO11, with replication of this finding within an independent cohort (Rye et al., 2011b). A previous study by Segade et al. (2006) also reported a nominal association at this locus. MacArthur et al. (2014) reported associations at the SMAD2 and SMAD4 loci, mediators of the TGF-β signalling pathway, along with associations at the TLR4 and MUC5B loci. These studies are consistent with our findings on the underlying genes and pathways involved with OME in the mouse models *Jf*, *Junbo* and *Tgif1*, each of which carry a mutation in genes known to be involved in the regulation of the TGF-β signalling pathway.

In addition to candidate-gene association studies, there have been two GWAS studies described (Rye et al., 2012; Allen et al., 2013). Rye et al. (2012) identified significant associations at several loci, including *CAPN14*, *GALNT14*, *BPIFA3* and *BPIFA1*, identifying novel candidate genes for further analysis. Interestingly, a mouse model of BPIFA1 was recently shown to demonstrate an increased susceptibility to OM (Bartlett et al., 2015). In a second GWAS study (Allen et al., 2013), a novel susceptibility locus on chromosome 2 was identified, with the relevant SNP lying in the intergenic region between *CDCA7* and *SP3*.

Overall, the reported association and GWAS studies underline the power of mouse models in identifying candidate genes and pathways, as well as validating the functionality of the human loci identified (Daly et al., 2004; Casselbrant et al., 2009; Rye et al., 2011a; MacArthur et al., 2014). In this study, we have focused on the *Fbxo11* gene, which is mutated in the OM model *Jf* (Hardisty-Hughes et al., 2006) and was identified as a potential OM susceptibility locus within two separate association studies (Segade et al., 2006; Rye et al., 2011b). We have now studied its genetic and biochemical interactions with p53 in order to elaborate its role within epithelia and its potential impact on the development of OM.

A variety of studies have demonstrated a crucial role for FBXO11 in the regulation of TGF-β signalling. *In vivo* studies of the *Jf* mutant have demonstrated markedly elevated levels of pSMAD2 associated with a variety of developmental epithelial abnormalities, including cleft palate, eyes open at birth and lung abnormalities (Tateossian et al., 2009). Recently, two important studies demonstrated a new role for FBXO11 in ubiquitin-dependent degradation of CDT2 that is crucial for TGF-β pathway responses, including cell cycle exit (Abbas et al., 2013a; Rossi et al., 2013). Moreover, p53 has been shown to be neddylated by FBXO11 *in vitro* (Abida et al., 2007) and, given the cross-talk between TGF-β and p53 signalling pathways, there is the potential for impacts on TGF-β signalling outputs via the interaction between p53 and FBXO11 *in vivo*. We surmised from our studies on the *Jf* mutant that aspects of embryonic epithelial development are controlled by FBXO11 through its regulation of the TGF-β pathway in cooperation with p53. Given the availability of FBXO11 and p53 mutants, we have undertaken a genetic approach to studying the pathways and mechanisms involved, including examining the genetic and biochemical interactions between these two factors. The middle ear and the airway epithelia have a number of biological properties in common (Takahashi, 2001), and therefore we have focused our studies on the developing lung.

Surprisingly, a detailed study of lung development in *p53* mutants identified an epithelial defect in a large proportion of homozygotes (around 40%). This phenotype had not been reported before and, although it was not fully penetrant, we observed a significant number of homozygous mutants with abnormal lung architecture showing a smaller number of airways of reduced size. This phenotype was associated with a very significant increase in the levels of pSMAD2 in the developing lung and a large increase in the number of epithelial cells positive for pSMAD2. These features replicate those found in the *Jf* mutant, although, in the *Jf* mutant, the penetrance of the lung phenotype is much higher. In the absence of p53 in the developing lung, we saw no effects on the protein expression of downstream genes, such as p21 and PAI-1. Cell line studies show that mutant p53 attenuates the ability of the TGF-β signalling pathway to induce the expression of genes such as p21 and PAI-1 (Cordenonsi et al., 2003).

We adopted various enhancements to investigating FBXO11-p53 interactions and found that, in contrast to our previous report, here we were able to show that FBXO11 interacts with and neddylates p53 in the developing lung. In *Jf* mice, mutant FBXO11 fails to interact with p53 and there is a significant reduction in neddylated p53, although there is no evidence for a corresponding increase in active p53. The *Jf* mutant, as with the *p53* mutant, shows raised levels of pSMAD2 (Tateossian et al., 2009). Moreover, in the presence of normal levels of p53, we find that levels of p21 and PAI-1 are significantly raised.

Given the similar developmental phenotypes shared by the *Jf* and *p53* mutants, we examined the phenotypes of double heterozygote *Jf/+ p53/+* mutant mice. The double heterozygotes showed defects in lung architecture, with extraordinary similarities to both *Jf* and *p53* homozygous mutants, including a reduced number of and smaller airways. The phenotypic outcome in the double mutant was not fully penetrant: only half of the double mutants were severely affected. The phenotypes observed in the double mutant demonstrate a genetic interaction between the *Fbxo11* and *p53* loci. Moreover, as with the *Jf* homozygote, we see increased levels of p21 and pSMAD2 in the double heterozygote compared to wild type. This implies a similar impact upon downstream pathways that could reflect a number of factors. These include lowered levels of FBXO11, which might be expected to lead to increased activation of p53, even with the lower levels of p53 found in the heterozygous state. However, it is clear that the effects of the *Fbxo11* and *p53* mutants are additive, and that the reduction in FBXO11 levels does not wholly compensate for the lower levels of p53 in the double heterozygote in terms of phenotypic outcomes.

Overall, we conclude that loss of function of either FBXO11 or p53 leads to developmental epithelial abnormalities in the lung that are associated with raised pSMAD2 levels. In addition, in double heterozygous mutants we also observe developmental epithelial abnormalities that are associated with significantly raised pSMAD2 levels. In the *Jf* mutant, where normal levels of p53 are maintained, we see significant increases in p21 and PAI-1 associated with the increases in pSMAD2, but this is not the case in the *p53* mutant, where p53 is absent. However, in the double heterozygote, where p53 is present, albeit at reduced levels, we also see significantly raised levels of p21. Taking the data together, we conclude that FBXO11 regulates the TGF-β pathway in the embryonic lung via cross-talk with p53 signalling.

Regulation of the TGF-β pathway by FBXO11 in the developing lung could reflect influences by the recently identified FBXO11-CDT2-SET8 pathway or via other routes, or a combination of both. We studied the levels of CDT2 and SET8 in the developing lung in both wild-type and mutant *Fbxo11* and *p53* mice. We found no significant change in the steady-state levels of these proteins in either mutant. Ubiquitin-dependent regulation of CDT2 by FBXO11 occurs via an SCF complex incorporating the cullin-1 RING ubiquitin ligase, and we confirmed in FBXO11 pull-downs that cullin-1 is present in developing lung tissue. It has been shown that the short isoform of FBXO11 binds cullin-1 and CDT2 and is crucial for CDT2 degradation (Abbas et al., 2013b). CDT2 is a substrate receptor for the cullin-4 ubiquitin ligase, promoting cell cycle progression through the degradation of SET8 and p21. Surprisingly, we found that an antibody that recognizes both the short and long isoforms of FBXO11, pulls down cullin-4, whereas an antibody recognizing the long isoform only does not. Moreover, this same antibody also pulls down CDT2. This suggests that the small form of FBXO11 (which does not interact with p53) binds to CDT2 within the cullin-4 RING ligase complex, as well as binding cullin-1. Despite the interactions between FBXO11 and CDT2 in the developing lung, there is no effect on levels of CDT2 or SET8. Moreover, the increase in levels of p21 seen in the *Jf* mutant cannot be explained by this pathway because knockdown of FBXO11 would be expected to lead to substantial reductions in the levels of p21 as well as SET8.

In conclusion, our results support the hypothesis that FBXO11 regulates the TGF-β pathway in the embryonic lung via cross-talk with p53, emphasizing the interplay of these two pathways and a crucial role of p53 in epithelial development, with consequent implications for the underlying pathological mechanisms of OM.

## MATERIALS AND METHODS

### Mice

*Jf* mutant mice were kept on a mixed C3H/HeH-C57BL/6J background because it was not possible to maintain them on a congenic C57BL/6J background. *p53* mice were maintained on a C57BL/6J background. The colonies were genotyped as previously described (Donehower et al., 1992; Hardisty-Hughes et al., 2006). All animal experimentation was approved by the Animal Welfare and Ethical Review Body at MRC, Harwell.

### Histology

Embryo samples were fixed in 10% buffered formaldehyde. Three-micrometre-thick paraffin sections were obtained and stained with haematoxylin and eosin for morphological assessment.

### Antibodies

The following primary antibodies were used: p53 (9282), pSMAD2 (3101), NEDD8 (2754), SET8 (C18B7), SUMO-1 (4930), Cell Signaling; p53 (FL-393 sc-6243), p21 (C-19; sc-397), PAI-1 (H-135; sc-8979), BCL-6 (N-3; sc-858), CUL1 (H-213 sc-11384) and CUL4 (H-66 sc-10782), Santa Cruz; FBXO11 (A301-178A and A301-177A), Bethyl Laboratories; MMP2 (RPCA-MMP2), EnCor Biotechnology Inc.; CDT2 (OAAB00993), AVIVA Systems Biology; pSMAD2 (AB3849), Millipore; actin (A 2066), Sigma. RAW 264.7 whole-cell lysate (sc-2211), Santa Cruz and A549 cell lysate, Cell Signaling were used as positive controls for the antibodies.

### Immunoprecipitation

The E15.5 embryos were removed from the uterus of the pregnant mice and transferred into cold PBS containing a cocktail of protease and phosphatase inhibitors (catalogue numbers 04 693 124 001 and 04 906 837 001; Roche). The lungs were then dissected out and the protein samples were prepared as previously described (Tateossian et al., 2009). For 2.5 mg total protein extract, 12.5 µg of antibody was used for the immunoprecipitation using Dynabeads protein G kit (10007D, Invitrogen) as described by the manufacturer. Rabbit IgG (P120-101 Bethyl Laboratories) was used as a negative control in IP experiments.

### Western blot

Tissue lysates were resolved in 4-12% or 12% NuPAGE Bis-Tris gels and immunoprecipitates in 7% NuPAGE Tris-Acetate gels (Invitrogen). They were all blotted onto nitrocellulose membrane (Invitrogen) and immunostained. Immunoprecipitates were assayed using ReliaBLOT kit (WB120, Bethyl Laboratories). Antibody dilutions for lung lysates were as follows: FBXO11, 1:2000; p53, 1:500; pSMAD2 (3101, Cell Signaling) 1:500; p21, 1:1000; PAI-1, 1:1000; MMP-2, 1:500; BCL-6, 1:1000; SET8, 1:500; NEDD8, 1:150; and CDT2, 1:250. Antibody dilutions for immunoprecipitates were: FBXO11, 1:400; p53, 1:25; CUL1, 1:100; and CUL4, 1:100. ECL detection system (GE Healthcare) was used.

### Immunohistochemistry

For immunohistochemical analysis of E15.5 lungs, the Vectastain Elite ABC kit (Vector Laboratories, PK 6101) was used according to the manufacturer's instructions. Sections were incubated with the anti-p53 antibody (1:100) or anti-pSMAD2 antibody (AB3849, Millipore; 1:200) overnight at 4°C. DAB+ chromogen system (DAKO K3468) was used to develop the specific signals and slides were counterstained with haematoxylin. Some sections were incubated with serum instead of primary antibody as negative controls.

### Data analysis

A chi-squared test was used to compare the difference between the observed and the expected number of *p53*-knockout mice and double-mutant mice. In other experiments, data was analyzed using unpaired two-tailed Student's *t*-tests. A *P*-value <0.05 was considered significant and *P*<0.001 highly significant.
